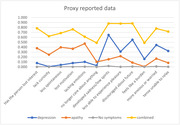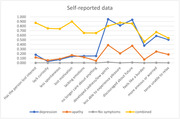# A Data‐Driven Examination of Apathy and Depression in Cognitively Normal Older Adults

**DOI:** 10.1002/alz.092258

**Published:** 2025-01-03

**Authors:** Miguel Vasconcelos Da Silva, Dag Aarsland, Clive G Ballard, Anne Corbett, Zahinoor Ismail, Byron Creese

**Affiliations:** ^1^ King’s College London, London United Kingdom; ^2^ University of Exeter, Exeter United Kingdom; ^3^ King’s College London, London, England United Kingdom; ^4^ University of Exeter, Exeter, Devon United Kingdom; ^5^ College of Medicine and Health, University of Exeter, Exeter United Kingdom; ^6^ Hotchkiss Brain Institute, University of Calgary, Calgary, AB Canada; ^7^ Brunel University London, London United Kingdom

## Abstract

**Background:**

Apathy and mood symptoms are increasingly recognised as clinical important aspects of prodromal dementia; both are associated with increased risk of dementia even in cognitively normal people. The clinical overlap between apathy and low mood poses a challenge in distinguishing between the two conditions. It is crucial to differentiate between depression and apathy, along with any underlying syndromes, to facilitate the development of targeted treatments. Using a data‐driven approach, we recently reported the existence of distinct apathy and depression clusters in dementia, confirming observations from the clinic and epidemiology. In this study we sought to establish whether similar patterns of symptoms were present in cognitively normal older adults

**Method:**

We analysed data from 21,925 community dwelling older adults. Latent class analysis (LCA) was applied to self‐reported and proxy ratings (obtained using the Mild Behavioral Impairment Checklist) of apathy and mood. Polygenic Risk Scores for Alzheimer’s disease (AD) and Major Depression (MDD) were tested for associated with class membership.

**Result:**

The LCA analysis using proxy data showed a 4‐class group which was considered the best model: No symptoms, Depression, Apathy/depression, and an Apathy group. The LCA using self‐reported data reveals the 4‐class group without a as a clear apathy class as the proxy data (see Figures 1 and 2). PRS for AD and MDD were only associated with depression and apathy/depression classes in self‐reported data (not in proxy data).

**Conclusion:**

This analysis highlights the apathy phenotype as a unique and separate condition, underscoring the imperative for additional research in this area. This emphasizes the potential for innovative approaches to delve deeper into the exploration and comprehension of apathy. The differences between the self and proxy reported data highlights the possibility of under reporting of apathy by patients.